# The structural origin of metabolic quantitative diversity

**DOI:** 10.1038/srep31463

**Published:** 2016-08-16

**Authors:** Seizo Koshiba, Ikuko Motoike, Kaname Kojima, Takanori Hasegawa, Matsuyuki Shirota, Tomo Saito, Daisuke Saigusa, Inaho Danjoh, Fumiki Katsuoka, Soichi Ogishima, Yosuke Kawai, Yumi Yamaguchi-Kabata, Miyuki Sakurai, Sachiko Hirano, Junichi Nakata, Hozumi Motohashi, Atsushi Hozawa, Shinichi Kuriyama, Naoko Minegishi, Masao Nagasaki, Takako Takai-Igarashi, Nobuo Fuse, Hideyasu Kiyomoto, Junichi Sugawara, Yoichi Suzuki, Shigeo Kure, Nobuo Yaegashi, Osamu Tanabe, Kengo Kinoshita, Jun Yasuda, Masayuki Yamamoto

**Affiliations:** 1 Tohoku Medical Megabank organization, Tohoku University, 2-1, Seiryo-machi, Aoba-ku, Sendai, 980-8573 Japan; 2 Graduate School of Medicine, Tohoku University, 2-1, Seiryo-machi, Aoba-ku, Sendai, 980-8575 Japan; 3 Graduate School of Information Sciences, Tohoku University, 6-3-09, Aramaki Aza-Aoba, Aoba-ku, Sendai, 980-8579 Japan; 4 Institute of Development, Aging and Cancer, Tohoku University, 4-1, Seiryo-machi, Aoba-ku, Sendai, 980-8575 Japan

## Abstract

Relationship between structural variants of enzymes and metabolic phenotypes in human population was investigated based on the association study of metabolite quantitative traits with whole genome sequence data for 512 individuals from a population cohort. We identified five significant associations between metabolites and non-synonymous variants. Four of these non-synonymous variants are located in enzymes involved in metabolic disorders, and structural analyses of these moderate non-synonymous variants demonstrate that they are located in peripheral regions of the catalytic sites or related regulatory domains. In contrast, two individuals with larger changes of metabolite levels were also identified, and these individuals retained rare variants, which caused non-synonymous variants located near the catalytic site. These results are the first demonstrations that variant frequency, structural location, and effect for phenotype correlate with each other in human population, and imply that metabolic individuality and susceptibility for diseases may be elicited from the moderate variants and much more deleterious but rare variants.

Metabolomics is emerging as an indispensable method for investigating the causes of diseases, because metabolite levels in biofluids are significantly influenced by various genetic and environmental factors. Recent technological advances have made it possible to implement the genome wide association study (GWAS) of metabolic traits to investigate genetic effects on blood metabolite levels^1^,^2^,^3^,^4^,^5^,^6^. Many blood metabolites appear to be associated with genetic loci, suggesting that normal blood metabolite levels may be influenced by genetic polymorphisms. In most of previous GWAS, individual samples were genotyped exploiting commercial array systems that contain a limited number of single nucleotide polymorphisms (SNPs), resulting in frequent difficulties in identifying the causal polymorphisms influencing the metabolite levels, leaving the question open as to how these polymorphisms affect the blood metabolite levels at a molecular or catalytic level.

As elucidation of the relationship between quantitative diversity of human blood metabolites and structural diversity of enzymes caused by variants in human population is critically important to understand the mechanisms how human metabolic individuality is defined, in this study we performed a large-scale cohort association study of metabolomics-genomics to investigate the relationship between structural variations in enzymes and metabolic phenotypes in human. We analyzed the metabolites of plasma collected from 512 participants in a population cohort conducted by Tohoku Medical Megabank organization (ToMMo) by nuclear magnetic resonance (NMR) spectroscopy. We obtained the metabolite profiling of plasma and analyzed the correlation among the quantified metabolites. We also performed an association study of plasma metabolites using whole-genome sequence dataset from all participants^7^ to elucidate true causal non-synonymous variants, instead of using SNP array data.

We identified five metabolites associated with non-synonymous variants in five metabolic enzymes, four of which were previously reported to be involved in metabolic diseases. To clarify the relationship between the variants and functional activity of related enzymes, we performed structural analysis of the five non-synonymous variants and found that they are not located in catalytic center regions, but located in peripheral regions or in regulatory domains, indicating that these variants retain only moderate impact on their corresponding enzymatic activities. Therefore, we further analyzed variants in these enzymes and found two individuals with larger changes of metabolite levels. These individuals have much more rare variants of one enzyme gene that cause non-synonymous variants located in closer proximity to the catalytic site, indicating that they cause larger functional impacts than the moderate variant. Whereas many studies have been conducted to clarify the relationship between gene variants and functional activities of proteins, our results unequivocally demonstrate that variant frequency, structural location, and effect for phenotype correlate with each other in human population. We expect that our approach is versatile to discover further associations of metabolites with diseases, as even a moderate variant can be detected as being associated with a significant change of plasma metabolite levels.

## Results and Discussion

### Design and operation of metabolome study

**Figure 1:**
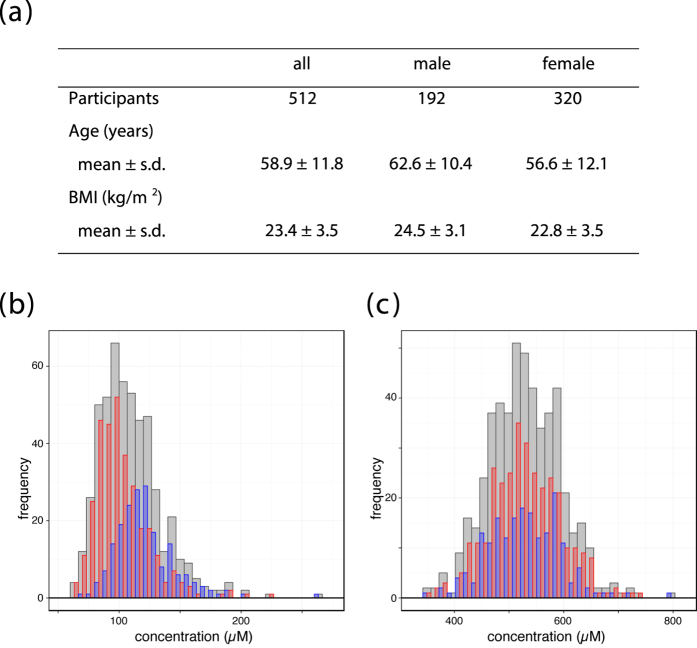
Study population. (**a**) Sample characteristics in this study. (**b**,**c**) Distributions of leucine (**b**) and glutamine (**c**) concentrations in plasma. Red and blue bars represent female and male, respectively.

Human plasma samples from 512 participants in the Community-Based Cohort Study executed by ToMMo were analyzed by using NMR spectroscopy. Sample characteristics in this study are summarized in Fig. 1a. We focused on hydrophilic low-molecular-weight metabolites, such as amino acids and their derivatives. As the concentrations of those metabolites are relatively low and there existed a possibility that some of the metabolites might interact with plasma proteins, metabolites were extracted from plasma for precise quantification. We identified and quantified 37 metabolites using the Chenomx NMR Suite software (Supplementary Table S1). Analyses of the concentration distribution of the metabolites identified variations of plasma metabolite levels in the healthy participants (Fig. 1b,c), demonstrating that the distributions of some metabolites are different between genders, whereas those of the other metabolites are not.

### Correlation analyses of plasma metabolites

**Figure 2:**
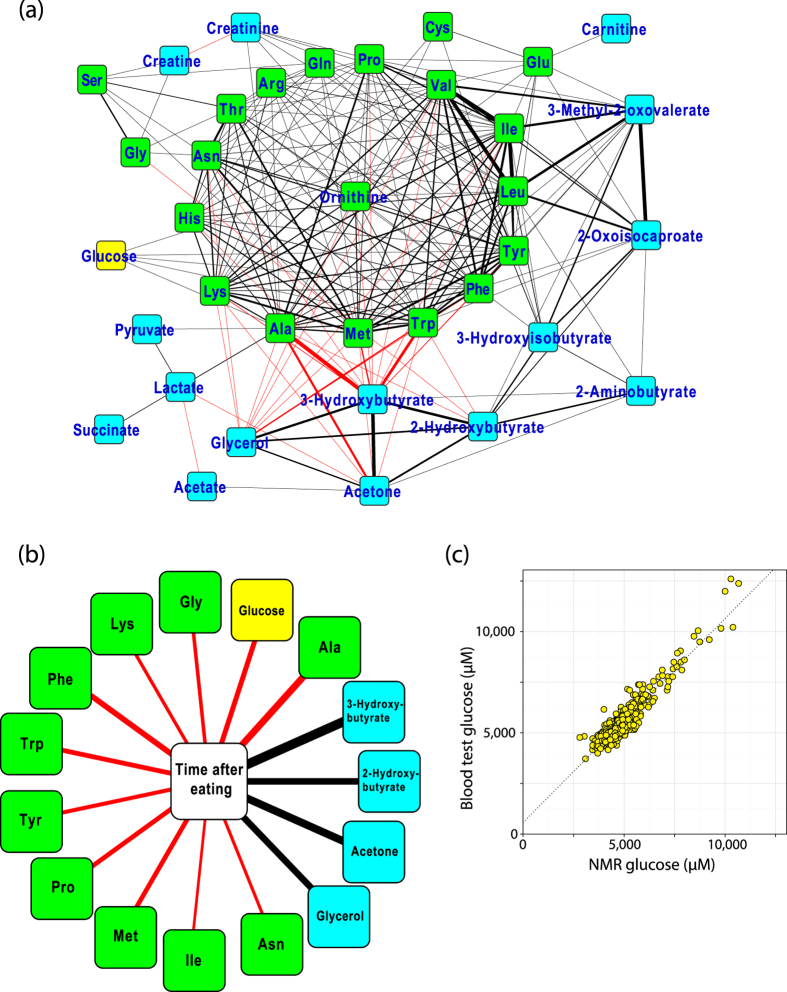
Correlation analyses. (**a**) Correlations between the quantified metabolites. Each node and each edge represent a metabolite and the metabolite pairs with log_10_(P-value) <−10, respectively. Positive and negative correlations are represented using black and red lines, respectively. Thicker lines represent stronger correlations between two metabolite levels. This figure was generated using Cytoscape v3.2.1^42^. (**b**) Correlations between time after eating and the quantified metabolites. Each node represents a metabolite or time after eating, and each edge represents the pairs with log_10_(P-value) <−10. Positive and negative correlations were represented same as (**a**). (**c**) Correlation between the plasma glucose concentration quantified by NMR and that from the blood test.

We conducted correlation network analyses of plasma metabolites, and found that most of the quantified metabolites are correlated with one another (Fig. 2a). The correlations between amino acids were positive (black lines), whereas those between amino acids and glycerol or ketone bodies were negative (red lines). Strong positive correlations were observed among leucine, isoleucine, valine, 3-mehtyl-2-oxovalerate, and 2-oxoisocaproate. These results reflect the networks involved in these metabolites, indicating that our quantified data are physiologically relevant.

We identified several metabolites correlated with time after eating in the questionnaire items (Fig. 2b). Ketone bodies and glycerol were positively correlated to time after eating (black lines), whereas amino acids and glucose were negatively correlated (red lines). These results reflect that plasma metabolite levels change in a time-dependent manner after nutrient intake and are consistent with the results of the correlation network analyses of the plasma metabolites (Fig. 2a), suggesting that plasma metabolite concentrations are good indicators for investigating the effects of diet on individual health conditions.

The concentration of plasma glucose quantified by NMR is highly correlated with the blood test value of plasma glucose, indicating the high precision of metabolite quantification by NMR (Fig. 2c). These results indicate that the quality of our cohort samples is high, and the accuracy of our metabolomics data is sufficient for the following association studies.

### Association study of metabolomics-genomics

**Figure 3:**
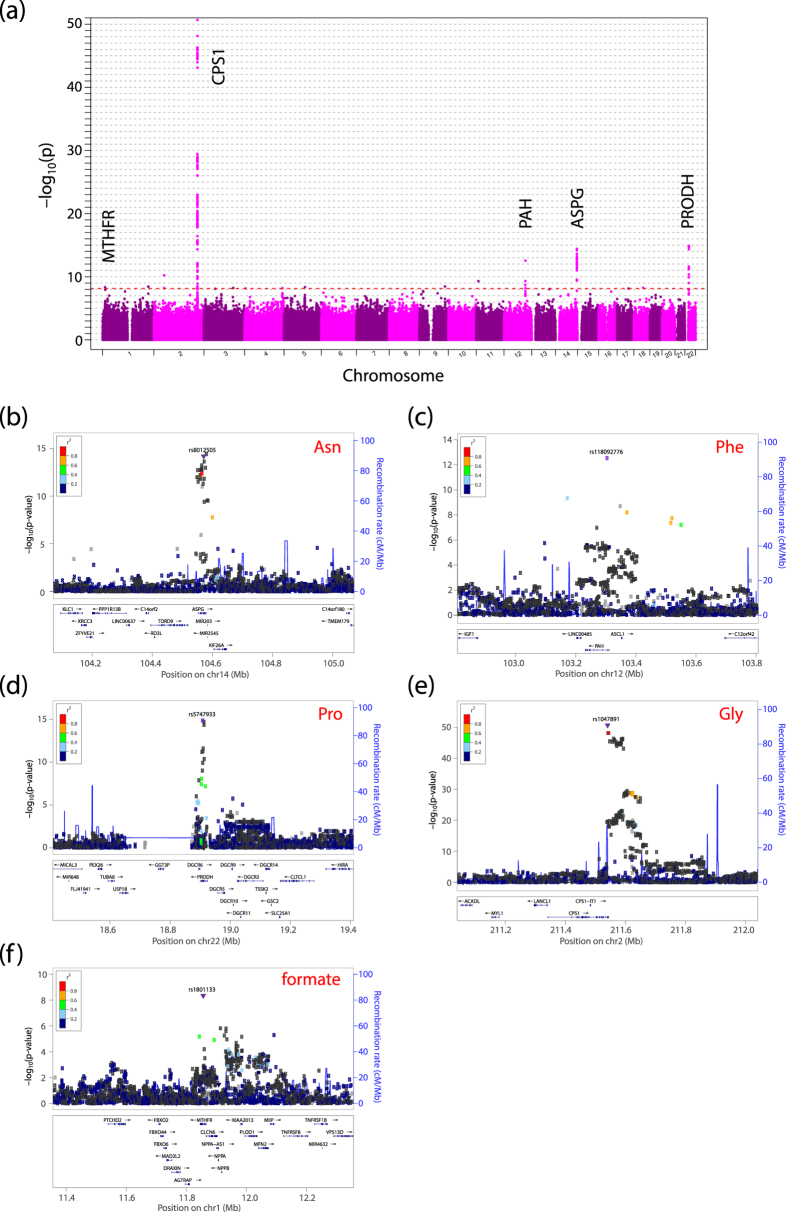
Association study of metabolomics and genomics. (**a**) Manhattan plots for metabolic traits. The strength of association with plasma metabolite concentrations for the five loci is shown based on the results from the association study for all 512 samples (Table 1). The line indicates a suggestive genome-wide significance level with a P-value of 7.08 × 10^−9^. (**b–f**) Regional association plots for significant loci reported in Table 1. Statistical Significance of associated SNPs is plotted on the −log_10_(P-value) scale as a function of chromosomal position (NCBI 37). The identified causal SNP at each locus is shown in purple. Correlation of the causal SNP to other SNPs at each locus is shown on a scale from minimal (blue) to maximal (red). Estimate recombination rate are also shown.

We performed association studies of the quantified plasma metabolites and SNPs data derived from the whole genome analysis of 512 cohort participants. We applied 512 high-resolution whole genome sequences^7^ for the analysis. As a result, we identified 5 genetic loci that significantly associated with plasma metabolite concentrations at a genome-wide significant P-value threshold (Table 1, Fig. 3, and Supplementary Fig. S1). Of note, these SNPs are located in coding regions and cause non-synonymous variants. Of these five SNPs causing non-synonymous variants, four are associated with amino acids, whereas one is associated with formate.

### Association of rs8012505 with asparagine

**Figure 4:**
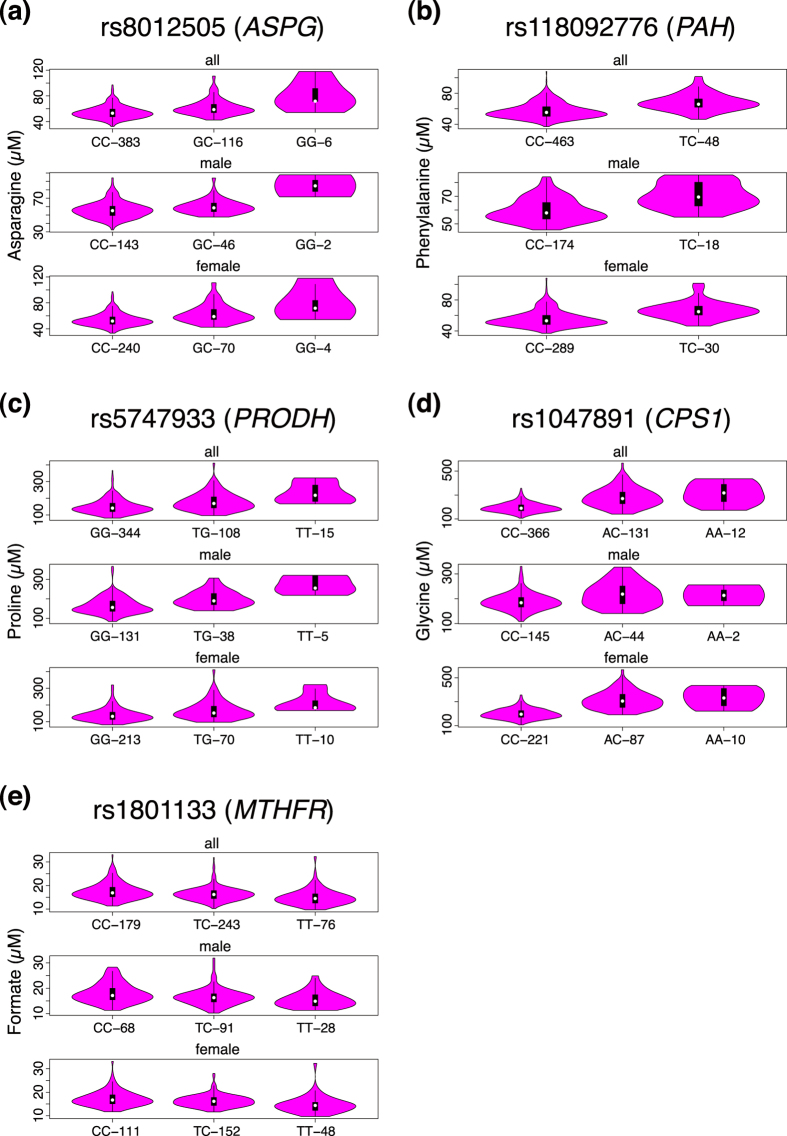
Distribution of the Plasma Metabolite Concentration. Distribution of the plasma metabolite concentration across the genotypes of each locus summarized in Table 1 was shown using a violin plot. These figures were made using R package.

One of the four SNPs associated with amino acids, rs8012505 is associated with asparagine, and this association has not been reported (Table 1, Fig. 3a,b, and Supplementary Fig. S1a). This SNP is located in the asparaginase gene (*ASPG*, also known as 60-kDa lysophospholipase), which product asparaginase (ASPG) catalyzes the hydrolysis of L-asparagine to L-aspartate and ammonia^8^. The rs8012505 causes non-synonymous variant S344R. This minor allele variant associates an increase of the asparagine concentration in plasma, suggesting that the S344R variant decreases the ASPG activity (Fig. 4a and Supplementary Table S2). In fact, heterozygotes of the SNP (116 cases) exhibited an average 13% increase of the plasma asparagine concentration compared with the wild-type homozygotes (383 cases), whereas the homozygotes of the variant allele (6 cases) exhibited a 48% increase in average. The association of this SNP to asparagine was significant for females, but not so significant for males (Supplementary Fig. S1a and Supplementary Table S3).

**Figure 5:**
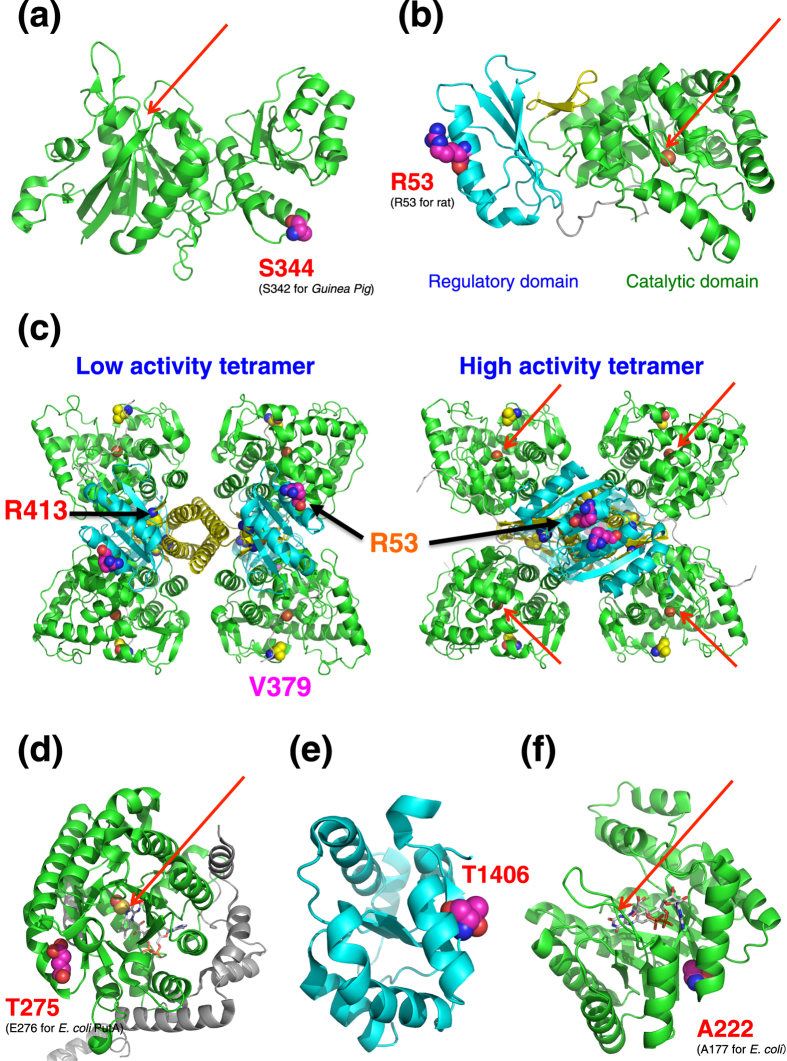
Mapping of the five non-synonymous variants on the reported crystal structures of the enzymes, respectively. Ribbon models of (**a**) structure of *Guinea pig* L-Asparaginase 1 Catalytic domain (Protein Data Bank (PDB) ID: 4R8 K), (**b**) structure of rat phenylalanine hydroxylase (PAH) (PDB ID: 2PHM), (**c**) two types of tetramer model structures of PAH, low activity tetramer and high activity tetramer, (**d**) structure of *E. coli* PutA proline dehydrogenase domain (PDB ID: 2FZM), (**e**) structure of human carbamoyl-phosphate synthase 1 (CPS1) regulatory domain (PDB ID: 2YVQ), and (**f**) structure of *E. coli* methylenetetrahydrofolate reductase (MTHFR) (PDB ID: 1B5T) are shown. The catalytic domains are depicted in green, whereas the regulatory domains are depicted in cyan. In the case of PAH structure, the C-terminal tetramerization domain is depicted in yellow. The residue corresponding to the position of each non-synonymous variant is shown by a sphere model. Cofactor FADs were represented by stick model for PRODH and MTHFR. The catalytic site of each enzyme is indicated by red arrow. The coordinates of the human PAH tetramers were obtained from the supplementary data of the reported article^15^. All figures were made using the PyMOL Software Package (https://www.pymol.org/).

We investigated structural basis of S344R variant. The structure of *guinea pig* ASPG shows that the Ser344 residue is located at the edge of the C-terminal region of the domain, away from the catalytic site (Fig. 5a)^8^,^9^. As human ASPG is known to be allosterically regulated by L-asparagine and putative asparagine binding site is located in the C-terminal region of the domain^8^, this variant likely influences the allosteric regulation of the enzyme to moderately reduce its activity.

Considering the association study for all (both male and female) cases, the P-value of another neighboring SNP rs61997624 (–logP value: 14.41) was slightly more significant than that of rs8012505. The SNP rs61997624 is located in an intergenic region downstream of *ASPG*, and the association is weaker than that of rs8012505 for females. On the other hand, the SNP rs4144027, located at more than 194-kb upstream of *ASPG*, was previously reported to be associated with asparagine^5^. We estimated the effects of these intergenic SNPs for the function of ASPG using a functional prediction program, CADD^10^. The CADD phred scores of rs61997624 and rs4144027 are 2.336 and 3.233, respectively, both of which are not significant values and are much lower than that of rs8012505 (15.87). These data indicate that our SNP rs8012505 is the true causative variant.

### Association of SNP rs118092776 with phenylalanine

We also identified an association between the SNP rs118092776 and phenylalanine (Table 1, Fig. 3a,c and Supplementary Fig. S1b). This SNP is located in the phenylalanine hydroxylase (*PAH*) gene and causes the non-synonymous variant R53H. PAH catalyzes hydroxylation of phenylalanine to tyrosine, a rate-limiting step in phenylalanine catabolism^11^. Severe deficiency of PAH activity causes hyper-phenylalaninemia (HPA), including phenylketonuria (PKU), the most severe phenotype of HPA. Our results demonstrate that the minor allele variant causes an increase in the phenylalanine concentration in plasma, indicating that this non-synonymous variant decreases the PAH activity (Fig. 4b; Supplementary Table S2). The heterozygotes of the SNP (48 cases) displayed an average 19% increase in plasma phenylalanine concentration compared with the wild-type homozygotes (463 cases). The homozygote of the variant allele was not identified in our samples because of its relatively low MAF (approximately 5%; Table 1).

Based on preceding structural analyses^12^, the R53H variant is located at the edge of the regulatory domain of PAH, indicating that it does not perturb directly the catalytic site, but may moderately perturb the allosteric regulation of PAH (Fig. 5b and Supplementary Fig. S2a). Alternatively, this variant may reduce the stability of the tetrameric and/or dimeric form of PAH^11^. Consistent with our current results, enzyme activity of the R53H mutant has been reported to decrease to 79%^11^,^13^, and compound heterozygote patients with this variant and other more severe PAH variants (as will be described in next section) have been reported to display a milder phenotype than compound heterozygotes of the severe variants^13^,^14^. Intriguingly, patients with the R53H allele were compensated with the administration of tetrahydrobiopterin (BH_4_), a cofactor of PAH^14^. Upon the addition of BH_4_, the PAH activity of R53H mutant increased from 63% to 139%, compared with wild type^11^, suggesting that the increased BH_4_ levels recovered the stability of the PAH dimer.

Although no structure of full length PAH tetramer has been reported, two architecturally different tetramers for PAH have been reported, *i.e.,* a high activity tetramer and a low activity tetramer, which are interconverted (Fig. 5c)^15^. In the high activity tetramer, two regulatory domains form a dimer and the Arg53 residue is located on the dimer interface. Moreover, recent biochemical study showed that the isolated regulatory domain of PAH exists in a monomer-dimer equilibrium and the binding of phenylalanine stabilizes the dimer^16^. Based on these observations, we surmise following scenario. The R53H variant may destabilize the dimer formation of the regulatory domains in the high activity tetramer that influences the regulation of PAH activity by phenylalanine, so that the R53H variant decreases the PAH activity.

### Association of other SNPs in 1KJPN with phenylalanine

Our results also identified some participants showing higher levels of plasma phenylalanine without the R53H variant (Fig. 4b). Since a number of non-synonymous variants besides R53H have been observed in *PAH* in HPA/PKU patients^13^,^14^,^17^,^18^,^19^,^20^, we investigated whether these non-synonymous variants are also present in the participants of our study with higher plasma phenylalanine levels.

**Figure 6:**
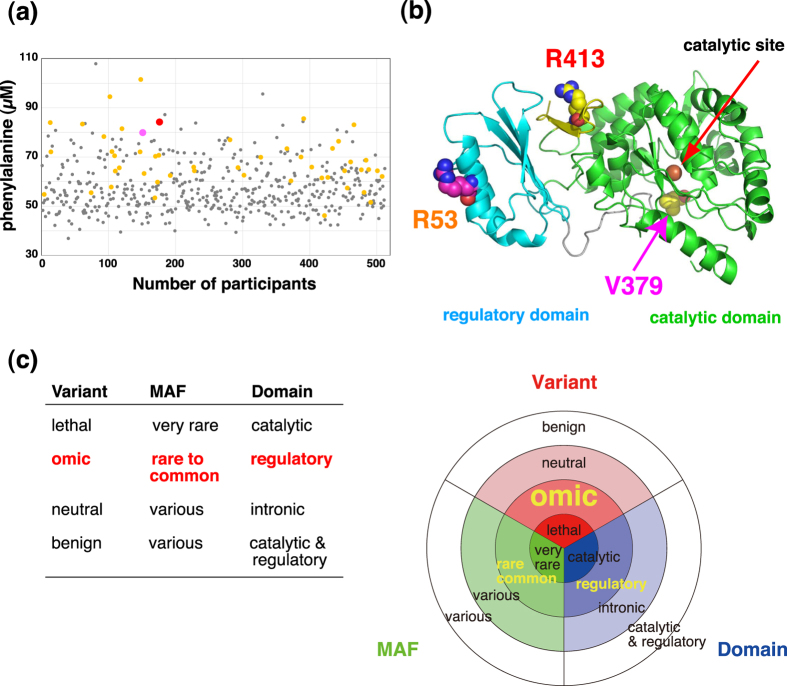
Relationship among variant, allele frequency, and structure. (**a**) Scatter plot of the concentration distribution of plasma phenylalanine. Heterozygotes of rs118092776, rs79931499, and rs746203167 are colored orange, red, and pink, respectively. Others are colored grey. (**b**) Mapping of the three non-synonymous variants on the structure of rat PAH. Three residues corresponding to the position of each non-synonymous variant are shown by a sphere model. (**d**) Schematic view of the relationship among variant, allele frequency, and structure. We propose a new category for variant, omic, whose variants partially influence omics environments of individuals.

We identified two participants harboring potentially pathogenic rare non-synonymous variants, R413P (SNP rs79931499) and V379A (SNP rs746203167). Both participants are heterozygotes, and their plasma phenylalanine levels were significantly higher than the average level (84.2 μM for R413P and 79.9 μM for V379A: Fig. 6a). Arg413 is located on the interface between the catalytic domain and the C-terminal tetramerization domain (Figs 6b and 5c, and Supplementary Fig. S2a), and is also placed near the regulatory domain of PAH, indicating that this variant actually affects the enzyme activity and/or stability of the protein complex. In contrast, Val379 is located near the catalytic site, suggesting that this variant directly affects the catalytic activity or destabilizes the enzyme (Figs 6b and 5c, and Supplementary Fig. S2a). These results unequivocally demonstrate that our approach is advantageous for identifying associations of plasma metabolite levels with rare variants, which cannot be identified by GWAS.

### Association of SNP rs5747933 with proline

We also detected association of plasma proline levels with rs5747933, a non-synonymous variant resulting in the T275N (isoform 1: T116N for isoform 2) substitution in proline dehydrogenase gene (*PRODH*) (Table 1, Fig. 3d, and Supplementary Fig. S1c). PRODH catalyzes the first step in proline catabolism, converting proline to ∆^1^-pyrroline-5-carboxylate (P5C). Deficiency of PRODH causes type I hyperprolinemia, manifesting high level of plasma proline, and is known to associate with schizophrenia^21^.

Our results revealed that this T275N variant causes an increase in plasma proline concentration, suggesting that the variant decreases the PRODH activity. As shown in Fig. 4c and Supplementary Table S2, the heterozygotes of the SNP (108 cases) exhibited an average 20% increase in plasma proline concentration compared with the wild-type homozygotes (344 cases) and this is statistically significant. Homozygotes of the variant allele (15 cases) exhibited an average 55% increase. Showing very good agreement, a recent exome-GWAS analysis also identified that this non-synonymous variant is associated with serum proline levels^6^.

The frequency of this variant has been reported to increase in type I hyperprolinemia patients compared with a control population, indicating that this variant is a probably pathologic variant. On the contrary, there is a report that this variant yielded not significantly detrimental effect on enzyme activity^21^. Structural analysis of *E. coli* PRODH shows that this variant is located at the edge of the dehydrogenase domain, away from the catalytic site (Fig. 5d)^22^. Interestingly, MTHFR (methylenetetrahydrofolate reductase), which contains the same TIM barrel fold and cofactor FAD as PRODH, is reported to be destabilized by a non-synonymous variant A222V at the edge of the domain, whereas K_m_ did not change by its variant^23^. Based on the observation, we envisage that the T275N variant also destabilizes the PRODH enzyme.

### Association of SNP rs1047891 with glycine

We also identified an association between rs1047891 and glycine (Table 1, Fig. 3e, and Supplementary Fig. S1d). This SNP is located in the carbamoyl phosphate synthetase 1 (*CPS1*) gene and causes the non-synonymous variant T1406N. The minor allele variant of this SNP increases the plasma levels of glycine (Fig. 4d and Supplementary Table S2). The heterozygotes of this SNP (131 cases) exhibited an average 44% increase in plasma glycine concentration compared with the wild-type homozygotes (366 cases), whereas the homozygotes of the variant allele (12 cases) exhibited an average 61% increase.

In liver mitochondria, CPS1 catalyzes the first step of the urea cycle, the conversion of ammonia and bicarbonate to carbamoyl phosphate, and its deficiency results in hyperammonemia, a metabolic disorder characterized by an excess of plasma ammonia levels^24^. It has been demonstrated that *CPS1* is associated with the blood levels of several metabolites, such as glycine and homocysteine, and affects the creatinine production and secretion function in chronic kidney disease^2^,^4^,^6^,^25^,^26^,^27^,^28^. Furthermore, the associations of *CPS1* T1406N variant with plasma glycine levels are much more significant for women than for men^29^, consistent with our current findings (Fig. 4d and Supplementary Fig. S1d). As for the effect of this variant on the catalytic activity of CPS1, there still remain some controversial arguments. One convincing evidence exploiting an *in vitro* enzyme assay supports the contention that this variant moderately reduced the enzymatic activity of CPS1^30^.

The mutated residue Thr1406 is located on the exposed surface of the C-terminal regulatory domain in CPS1, away from the catalytic site of this enzyme (Fig. 5e and Supplementary Fig. S2b), indicating that this variant does not influence the catalytic site itself but modulates the regulatory mechanism of the CPS1 enzyme^31^.

The mechanism underlining the increase of plasma glycine levels caused by this variant remains to be elucidated. One plausible hypothesis is to assume contribution of the glycine cleavage system for this association^28^. The glycine cleavage system consists of four components bound to the mitochondrial membrane and converts glycine to ammonia, bicarbonate, and methylene groups in a reversible manner^32^. The elevated level of ammonia caused by the CPS1 variant appears to shift the equilibrium reaction of the glycine cleavage system from the ammonia synthesis to glycine accumulation (Supplementary Fig. S3). Glycine is a source of creatinine, one of the metabolites associated with the CPS1 variant. In fact, plasma levels of glycine, serine, and creatine (precursor of creatinine) in healthy people are correlated with one another (Fig. 2a). Hence, this CPS1 variant seems to activate the creatinine synthesis pathway for the excretion of glycine.

### Association of SNP rs1801133 with formate

Additionally, we identified an association between formate and a *MTHFR* gene variant (SNP rs1801133) causing the non-synonymous variant A222V (Table 1, Fig. 3f, and Supplementary Fig. S1e). The minor allele variant of this SNP caused decrease in plasma formate concentration (Fig. 4e and Supplementary Table S2). The heterozygotes of the SNP (243 cases) exhibited an average 7% decrease in plasma formate concentration compared with the wild-type homozygotes (179 cases), whereas the homozygotes of the variant allele (76 cases) exhibited an average 15% decrease. Individuals homozygous for the valine substitution display approximately 30% of the normal enzyme activity, whereas heterozygotes display approximately 65% of the normal enzyme activity compared with individuals with the wild type alanine residue^33^.

This SNP is associated with many types of cancer and cardiovascular disease, and individuals with the homozygous variant have elevated plasma homocysteine levels^26^,^33^,^34^. The *E. coli* MTHFR structure shows that this mutated residue is located at the edge of the structure, out of the catalytic site (Fig. 5f)^23^. Guenther *et al*. also investigated the effect of the A177V (A222V for human MTHFR) variant on MTHFR and demonstrated that this variant did not affect K_m_ and k_cat_ but resulted in a thermolabile enzyme that loses its FAD cofactor more readily than does the wild type. These results indicate that this variant does not affect the catalytic site directly, but destabilizes the enzyme. Although the biological relationship between MTHFR and formate is unclear, formate is one of the one-carbon sources in folate metabolism and a precursor for the formation of 10-formyl-THF, indicating that the reduced activity of MTHFR observed with the A222V variant facilitates the consumption of formate as a one-carbon source.

### Association with creatinine

Finally, we investigated the association of metabolites with SNPs in non-coding regions, using whole genome sequencing data. We found that several SNPs in non-coding regions were probably associated with metabolites, although biological significance of these associations was not clear (data not shown). Hence, we selected the SNPs whose biological functions were already reported. Among these SNPs, we identified that one SNP rs820336 was associated with plasma creatinine levels, although not at a genome-wide significance level (Supplementary Fig. S1f and Supplementary Table S3). This association seems to be obvious only for female. The SNP rs820336 is located in an intron of the myosin light chain kinase (*MYLK*) gene. The substitution of G to A at rs820336 increased the plasma creatinine levels (Supplementary Table S2 and Supplementary Fig. S4). This variant is reported to associate with inflammatory lung disease and the substitution resulted in a significant decrease in the promoter activity of smooth muscle MLCK (smMLCK, encoded by *MYLK*)^35^. We also found that another SNP rs33262, also located in an intron of *MYLK*, has a slightly lower P-value compared with the case of rs820336, though whether rs33262 regulates *MYLK* or not remains to be elucidated.

### Power of combination of NMR metabolomics and GWAS

It has been demonstrated in several cohort studies that the combination of NMR-based metabolite analysis with GWAS is a robust way to identify loci influencing blood metabolite levels^3^,^6^. Of the studies, following two studies are seminal. Investigation of the association of 7.7 million SNPs with 216 serum metabolite phenotypes (117 metabolites and 99 variables derived from these measured metabolites) on 8330 Finnish individuals revealed that 31 loci are associated with one or more phenotypes^3^. Similarly, investigation for the pairs of 42 serum metabolites and 2.5 million SNPs on 2482 individuals identified 8 loci that are associated with metabolites^6^. The latter team expanded their study and performed exome sequence analysis for these 8 loci in 921 individuals, and identified potentially causal variants located in three genes, two of which are identical with those identified in our study.

Of note, while these studies directly measured serum metabolite levels without extraction and identified not only low-molecular weight metabolites but also many types of lipids and/or lipoproteins, in this study we measured plasma metabolite levels with an extraction protocol and quantified only hydrophilic metabolites. Although the direct measurement approach has an advantage to examine serum levels of lipids and lipoproteins, it is difficult to measure low-molecular weight metabolites bound to proteins or relatively low concentration metabolites, because the signals from these metabolites are suppressed by the effect of NMR pulse sequence or are overlapped with residual large protein/lipid signals. The direct measurement approach and extraction approach give rise to substantial difference. For instance, we have quantified 19 of the 20 standard amino acids, whereas the direct measurements were able to analyze only 8 and 12 amino acids^3^,^6^, respectively.

The latter study has demonstrated that the combination of GWAS and exome sequence analyses is quite effective for identifying causal variants compared with GWAS alone^6^, and presented advancement from the studies based on the GWAS. While exome sequence analysis is less expensive than whole genome sequence analysis, the latter provides almost complete sequence information of the genome, indicating that the approach applied in this study seems to be more effective for identifying causal variants.

In order to confirm the results of our association study, we performed a replication study using the other 230 samples (122 for male and 108 for female). We found that the five missense variants are clearly associated with respective metabolites (data not shown). In the case of the regulatory variant (*i.e.,* MYLK rs820336 for creatinine), the effect of the variant appears not to be clear, perhaps because this association is detected only for female but the number of female samples used is not enough. We are planning to conduct a more detailed replication study after finishing our cohort data cleaning process.

### Inter-ethnic and gender differences in the allele frequencies of non-synonymous variants

There are inter-ethnic differences in the allele frequencies of all non-synonymous variants identified in this study (Table 1). For example, the CPS1 T1406N variant is observed at a MAF of 30% in Caucasians, but at a MAF of 15% in Japanese (Table 1). Conversely, the PRODH T275N and MTHFR A222V variants are observed more frequently in East Asians (Japanese and Chinese) than in Caucasians and Africans. Notably, the PAH R53H variant is only found in East Asians. These results suggest that some plasma metabolite levels are significantly different between ethnic groups.

Our results also demonstrate that the effects of these genotype variations on plasma metabolite levels are different by gender (Supplementary Fig. S1 and Supplementary Table S3). For example, the increase of plasma glycine levels associated with the CPS1 T1406N variant is much more significant in females, consistent with the previous observation^29^. The effect of the MYLK variant on plasma creatinine levels was also significant only in females. These results demonstrate that the ethnic and gender differences significantly influence plasma metabolite levels.

### Prediction of the effects of genetic variants for enzyme function

In this study, we estimated the effects of the six genetic variants for the function of each enzyme using four distinct functional prediction programs (Table 2)^10^,^36^,^37^,^38^. These prediction programs produced similar results in predicting that the three non-synonymous variants, ASPG S344R, PAH R53H, and MTHFR A222V, are more damaging, whereas the effects of the other two non-synonymous variants, CPS1 T1406N and PRODH T275N, are less damaging. The PAH R53H variant decreases the enzyme activity, whereas MTHFR A222V variant destabilizes the enzyme. Whether the ASPG S344R variant affects its enzymatic activity has not been reported, our results suggest that this variant decreases its enzymatic activity.

Intriguingly, while the CPS1 T1406N variant was classified as “benign” or “tolerated” by the three programs, our results and previous reports clearly demonstrate that this variant is associated with plasma glycine and homocysteine levels, indicating that it moderately affects CPS1 function. Although the PRODH T275N variant is predicted to be tolerated by the Provean and SIFT programs, this variant is associated with plasma proline levels in our and previous GWAS^6^. Frequency of this variant is reported to be increased in type I hyperprolinemia patients compared with a control population^21^. Therefore, we would like to propose that this variant also destabilizes the enzyme and combinations of this variant with other more rare *PRODH* variants results in more severe phenotypes. These results indicate that other variants predicted as “tolerated” by these programs may also significantly affect individual metabolite properties.

### The structural origin of metabolic quantitative diversity

As shown in Fig. 5 and Supplementary Fig. S2, we have mapped the five non-synonymous variants onto the tertiary structures of the associated enzymes. Our results clearly show that these variants are located not in the catalytic sites, but in the peripheral regions of the catalytic or regulatory domains. We surmise that these variants perhaps destabilize the enzymes and/or affect their allosteric regulations, rather than directly perturbing their catalytic sites. These results indicate that only variants moderately affecting their enzymatic activities are commonly observed in healthy people. On the other hand, two PAH rare variants, which caused higher phenylalanine levels in plasma, are located closer to the catalytic sites than the common R53H variants (Fig. 6a,b), indicating that the variants in catalytic sites are normally too serious to be inherited. These results are the first demonstrations that the variant frequency, structural location, and effect for metabolic phenotype are correlated with each other in human population. Previous thought that allele frequency and fraction of occurrence in structurally and functionally important regions are correlated for non-synonymous SNPs^39^ shows very good agreement with our current results.

Variants moderately affecting the enzymatic activity or ‘moderate variants’ seem to be nearly neutral in their effects on individual fitness, resulting in their accumulation during evolution. We suggest that the accumulations of the moderate variants significantly influence the metabolic individuality and susceptibility for diseases. In fact, the variants identified in this study are also involved in a variety of diseases reported in the literatures. Combinations of these moderate variants in the different enzymes in the closely-related metabolic pathways, particularly those in the same pathway, may significantly increase the risk of many types of diseases. As shown in Fig. 6c, we propose that these variants are grouped into a new category “omic”, whose variants are rare to common and mainly found in regulatory domains of enzymes. We suggest that these “omic” variants are one of the main players that influence the metabolic individuality and susceptibility for diseases. Because our present approach can detect these moderate variants as a significant change in metabolite profiles, further studies will reveal unexpected associations of metabolites with diseases.

## Conclusions

We performed a Japanese population-based cohort metabolome study combined with whole genome sequence analysis. Our results demonstrate that the plasma levels of five metabolites are significantly associated with genetic polymorphisms causing non-synonymous variants. All five genes encode enzymes directly involved in metabolic pathways, and four of these variants are known to be associated with a variety of human diseases. Our results clearly show that variants moderately affecting their enzymatic activities are detected in healthy people. These variants seem to be nearly neutral and partially affect omics environment of individuals, such as metabolites. These variants are grouped into a new category “omic”, which are one of the main players that influence the metabolic individuality and susceptibility for diseases.

## Methods

### Study Population

Our cohort study in the Tohoku Medical Megabank Project is a population-based cohort for 150,000 people living in Miyagi-prefecture and Iwate-prefecture, a north region of Japan. The participants in this study were not selected based on any outcome or disease. We selected 512 adult people (ages above 20 years) whose whole genome sequences were already sequenced in our previous study (Whole-genome reference panel of Japanese (1KJPN) in Tohoku Medical Megabank organization)^7^. All experimental protocols of our cohort and metabolome studies were approved by the Ethics Committee of Tohoku University. Our studies were carried out in accordance with the approved guidelines. The written informed consent was obtained from all subjects.

### Sample Preparation

Blood samples were collected from 512 cohort participants using vacutainer tubes containing EDTA-2Na (Venoject II, Terumo Corporation). Blood was immediately stored at 4 °C and transported to the ToMMo BioBank laboratory. Plasma was prepared and stored at −80 °C using MATRIX^®^ 2D screw tube (Thermo Scientific). Metabolites were extracted using a standard methanol extraction procedure using 200 μL of plasma per sample. 4-times volume of 100% cold methanol (−30 °C) was added to plasma and the solution was mixed for 2 min, incubated for 10 min on ice, and centrifuged at 15,000 g for 10 min at 4 °C. The supernatant was transferred to a new tube and evaporated. Each dried sample was suspended in a 200 μL solution of 100 mM sodium phosphate buffer (pH 7.4) in 100% D_2_O containing 200 μM d_6_-DSS. 190 μL of solution was transferred to a 3 mm Bruker SampleJet NMR tube.

### NMR spectroscopy

All NMR experiments were performed at 298 K on a Bruker Avance 600 MHz spectrometer equipped with a CryoProbe and a SampleJet sample changer. Standard 1D NOESY and CPMG (Carr-Purcell-Meiboom-Gill) spectra were obtained for each plasma sample^40^. All spectra were acquired with 64 scans and 32 k of complex data points. All data were processed using the Chenomx NMR Suite 8.0 processor module (Chenomx). Metabolites were identified and quantified using the target profiling approach implemented in the Chenomx Profiler module. Standard 1D NOESY spectra were analyzed for the identification and quantification of metabolites. 1D CPMG spectra were also used for eliminating the influence of residual proteins to the quantification. To confirm metabolite identifications, 2D TOCSY and ^1^H,^13^C-HSQC experiments were collected for several samples. Spiking experiments were also performed to confirm the identifications.

### Statistical analysis

Spearman correlations between all pairs of metabolite concentrations were calculated by the R Hmisc package^41^. Correlations were illustrated as a network using Cytoscape v3.2.1^42^. Node positions were initially determined by using the Organic layout algorithm in Cytoscape v3.2.1 and manually modified for convenience. Spearman correlations between metabolite concentrations and time after eating (<10 hours) were also calculated by R and illustrated as a network using Cytoscape. Correlation analysis was performed on 240 individuals whose times after eating were less than 10 hours because values more than 10 hours were categorized into only one group in the questionnaire. Finally, Spearman correlation between the plasma glucose concentration quantified by NMR and that from the blood test was also calculated using R.

### GWAS analysis with whole-genome sequence data

From the 27,490,104 pre-filtering SNVs of the 1KJPN reference panel with 1,070 Japanese individuals^7^, we extracted 512 individuals with NMR experimental results. To construct the high-confidence SNVs, we have generated the sequencing data from HiSeq 2500 sequencers with a PCR-free protocol with 32.4x coverage and calculated the variants using the alignment tool Bowtie2 (version 2.1.0) with the variant caller Bcftools (ver. 0.1.17-dev)^43^,^44^. GRCh37/hg19 with the decoy sequence (hs37d5) was used as the human reference genome^7^. We divided the 512 individuals into a male and female dataset with 192 and 320 samples, respectively. We have applied genome wide association studies (GWASs) to NMR data for these three datasets after removing SNVs with the following conditions; the SNVs overlapping insertion or deletion detected by HaplotypCaller implemented in the Genome Analysis Toolkit (version 2.5–2) in the previous study^7^ were excluded from this analysis, minor allele frequency <0.01, P-value of the Hardy-Weinberg equilibrium test <0.0001, and missing genotype rate >0.1. After filtering, the numbers of variants for the datasets with male samples, female samples, and both samples were reduced from 14,702,219 to 7,338,054, from 17,524,867 to 7,108,834, and from 20,713,670 to 7,059,372, respectively. In the GWAS, an additive linear regression model adjusted with BMI and age was considered, and the P-value for each variant was obtained from t-tests on coefficients for its corresponding alleles using PLINK1.9 with the -linear option^45^. According to the Bonferroni correction, the genome-wide significance level for each dataset was set to 0.05 divided by the number of variants in the dataset, i.e., male dataset (6.81 × 10^−9^), female dataset (7.03 × 10^−9^), and both dataset (7.08 × 10^−9^).

## Additional Information

**How to cite this article**: Koshiba, S. *et al*. The structural origin of metabolic quantitative diversity. *Sci. Rep.* **6**, 31463; doi: 10.1038/srep31463 (2016).

## Supplementary Material

Supplementary Information

## Figures and Tables

**Table 1:** Genome-wide significant loci associated with metabolites.

Metabolites	sex	−log P	SNP	Chr.	Gene	Allele	MAF	Residue change
asparagine	all	14.19	rs8012505	14	*ASPG*	C > G	0.127	S344R
phenylalanine	all	12.54	rs118092776	12	*PAH*	C > T	0.047	R53H
proline	all	14.88	rs5747933	22	*PRODH*	G > T	0.148	T275N
glycine	all	50.66	rs1047891	2	*CPS1*	C > A	0.152	T1406N
formate	all	8.34	rs1801133	1	*MTHFR*	C > T	0.397	A222V
Chr., chromosome; MAF, minor allele frequency								
**MAFs of SNPs investigated in this study**								
			MAF					
Gene			*ASPG*	*PAH*	*PRODH*	*CPS1*	*MTHFR*	*MYLK*
Allele			C > G	C > T	G > T	C > A	C > T	G > A
Non-synonymous variant			S344R	R53H	T275N	T1406N	A222V	
ToMMo			0.127	0.047	0.148	0.152	0.397	0.020
CSHL- HAPMAP	HapMap-JPT	Japan	0.852^*1^			0.153	0.360	0.017
	HapMap-HCB	China	0.909^*1^			0.159	0.512	0.058
	HapMap-CEU	European (Utah, USA)	0.817^*1^			0.295	0.310	0.808
	HapMap-YRI	Sub-Saharan African	0.567^*1^			0.283	0.093	0.044
	HAPMAP-ASW	African ancestry in Southwest USA.				0.388	0.112	0.214
1000 GENOMES (pilot 1)	YRI_low_coverage_panel		0.051			0.186	0.119	0.008
	CEU_low_coverage_panel		0.175		0.083	0.300	0.275	0.800
	CHB + JPT_low_coverage_panel		0.117	0.025	0.158	0.100	0.433	0.042

MAFs of HAPMAP and 1000GENOMES are obtained from NCBI dbSNP database (http://www.ncbi.nlm.nih.gov/SNP/).

*1: Definition of the minor allele for ASPG in CSHL-HAPMAP seems to be opposite to that in 1000GENOMES or our study.

**Table 2:** Prediction of functional effects of the SNPs.

Metabolites	SNP	Gene	Allele	Residue change	CADD		polyphen 2		Provean		SIFT	
					impact	phred score	prediction	score	prediction (cutoff = 0.05)	score	prediction	score
asparagine	rs8012505	ASPG	C > G	S344R	Moderate, modifier	15.87	probably damaging	0.988	Damaging	0.016	Damaging	0.03
proline	rs5747933	PRODH	G > T	T275N	Moderate, modifier	7.274	no data		Tolerated	0.636	Tolerated	0.5
phenyl- alanine	rs118092776	PAH	C > T	R53H	Moderate, modifier	17.68	no data		Damaging	0.007	Damaging	0.01
glycine	rs1047891	CPS1	C > A	T1406N	Moderate, modifier	14.03	benign	0.009	Tolerated	0.235	Tolerated	0.25
formate	rs1801133	MTHFR	C > T	A222V	Moderate	21.9	probably damaging	0.998	Deleterious	−3.76	Damaging	0.05
creatinine	rs820336	MYLK	G > A	intron	Modifier	4.782						
