# Supplementary material for: The structural origin of metabolic quantitative diversity
Source: Sci Rep. 2016 Aug 16;6:31463. doi: 10.1038/srep31463 (PMC4985752; doi:10.1038/srep31463)
Supplement: Supplementary Information [file srep31463-s1.pdf]

The structural origin of metabolic quantitative diversity

Seizo Koshiba, Ikuko Motoike, Kaname Kojima, Takanori Hasegawa, Matsuyuki Shirota, Tomo Saito, Daisuke Saigusa, Inaho Danjoh, Fumiki Katsuoka, Soichi Ogishima, Yosuke Kawai, Yumi Yamaguchi-Kabata, Miyuki Sakurai, Sachiko Hirano, Junichi Nakata, Hozumi Motohashi, Atsushi Hozawa, Shinichi Kuriyama, Naoko Minegishi, Masao Nagasaki, Takako Takai-Igarashi, Nobuo Fuse, Hideyasu Kiyomoto, Junichi Sugawara, Yoichi Suzuki, Shigeo Kure, Nobuo Yaegashi, Osamu Tanabe, Kengo Kinoshita, Jun Yasuda, & Masayuki Yamamoto

Supplementary Materials

CONTENTS

SUPPLEMENTARY TALBLE
Supplementary Table S1.
Supplementary Table S2.
Supplementary Table S3.

SUPPLEMENTARY FIGURE
Supplementary Figure S1.
Supplementary Figure S2.
Supplementary Figure S3.
Supplementary Figure S4.

Supplementary Table S1: Plasma metabolites identified and quantified in this study

Metabolite	mean ± s.d. (μM)	Metabolite	mean ± s.d. (μM)
2-Aminobutyrate	16.7 ± 5.7	Glycerol	78.4 ± 29.0
2-Hydroxybutyrate	34.7 ± 15.8	Glycine	220.2 ± 68.4
2-Oxoisocaproate	24.4 ± 6.2	Histidine	79.1 ± 10.6
3-Hydroxybutyrate	95.2 ± 125.0	Isoleucine	57.9 ± 17.7
3-Hydroxyisobutyrate	12.1 ± 3.8	Lactate	2222.7 ± 605.6
3-Methyl-2-oxovalerate	18.4 ± 4.3	Leucine	109.3 ± 25.8
Acetate	37.3 ± 9.0	Lysine	119.9 ± 23.7
Acetone	7.9 ± 7.0	Methionine	23.5 ± 6.1
Alanine	331.8 ± 80.7	Ornithine	63.2 ± 15.8
Arginine	62.2 ± 15.6	Phenylalanine	58.2 ± 10.5
Asparagine	56.5 ± 11.5	Proline	158.5 ± 48.4
Carnitine	49.6 ± 10.3	Pyruvate	73.8 ± 17.1
Creatine	33.3 ± 17.3	Serine	114.7 ± 21.5
Creatinine	55.1 ± 19.6	Succinate	6.9 ± 1.3
Cysteine	52.2 ± 17.3	Threonine	132.3 ± 29.3
Formate	16.6 ± 3.3	Tryptophan	49.0 ± 9.1
Glucose	4844.2 ± 994.2	Tyrosine	64.8 ± 14.9
Glutamate	53.6 ± 16.6	Valine	209.2 ± 43.5
Glutamine	528.6 ± 65.4		

Supplementary Table S2: average values of metabolites

				Average			Fold change	
Meabolite	SNP	gene	sex	wt	hetero	homo	hetero/wt	homo/wt
asparagine	rs8012505	*ASPG*	all	54.6	61.8	80.8	1.13	1.48
			male	56.1	59.8	84.7	1.07	1.51
			female	53.8	63.1	78.9	1.17	1.47
phenylalanine	rs118092776	*PAH*	all	57.2	68.2	N.A.	1.19	N.A.
			male	59.9	70.9	N.A.	1.18	N.A.
			female	55.6	66.6	N.A.	1.2	N.A.
proline	rs5747933	*PRODH*	all	149.5	179.0	231.1	1.2	1.55
			male	165.3	199.0	271.4	1.2	1.64
			female	139.9	168.2	210.9	1.2	1.51
Glycine	rs1047891	*CPS1*	all	194.8	280.4	313.6	1.44	1.61
			male	187.8	217.5	213.5	1.16	1.14
			female	199.4	312.2	333.6	1.57	1.67
formate	rs1801133	*MTHFR*	all	17.6	16.4	15.0	0.93	0.85
			male	18.1	16.6	15.5	0.92	0.86
			female	17.2	16.3	14.7	0.95	0.85
creatinine	rs820336	*MYLK*	all	54.9	61.5	N.A.	1.12	N.A.
			male	66.9	63.2	N.A.	0.94	N.A.
			female	47.6	60.3	N.A.	1.27	N.A.

Supplementary Table S3a: Genome-wide significant loci associated with metabolites

Metabolite	sex	–log P	n	SNP	Chr.	Gene	Allele	MAF	Residue change
asparagine	all	14.19	505	rs8012505	14	*ASPG*	C>G	0.127	S344R
proline	all	14.88	467	rs5747933	22	*PRODH*	G>T	0.148	T275N
phenylalanine	all	12.54	511	rs118092776	12	*PAH*	C>T	0.047	R53H
glycine	all	50.66	509	rs1047891	2	*CPS1*	C>A	0.152	T1406N
formate	all	8.34	498	rs1801133	1	*MTHFR*	C>T	0.397	A222V

Supplementary Table S3b: Loci associated with metabolites (sex specific)

Metabolite	sex	–log P	n	SNP	Chr.	Gene	Allele	MAF	Residue change
asparagine	female	11.23	314	rs8012505	14	*ASPG*	C>G		S344R
glycine	female	44.64	317	rs1047891	2	*CPS1*	C>A		T1406N
creatinine	female	6.90	316	rs820336	3	*MYLK*	G>A	(0.020 for all)	intron

(a) Asparagine rs8012505

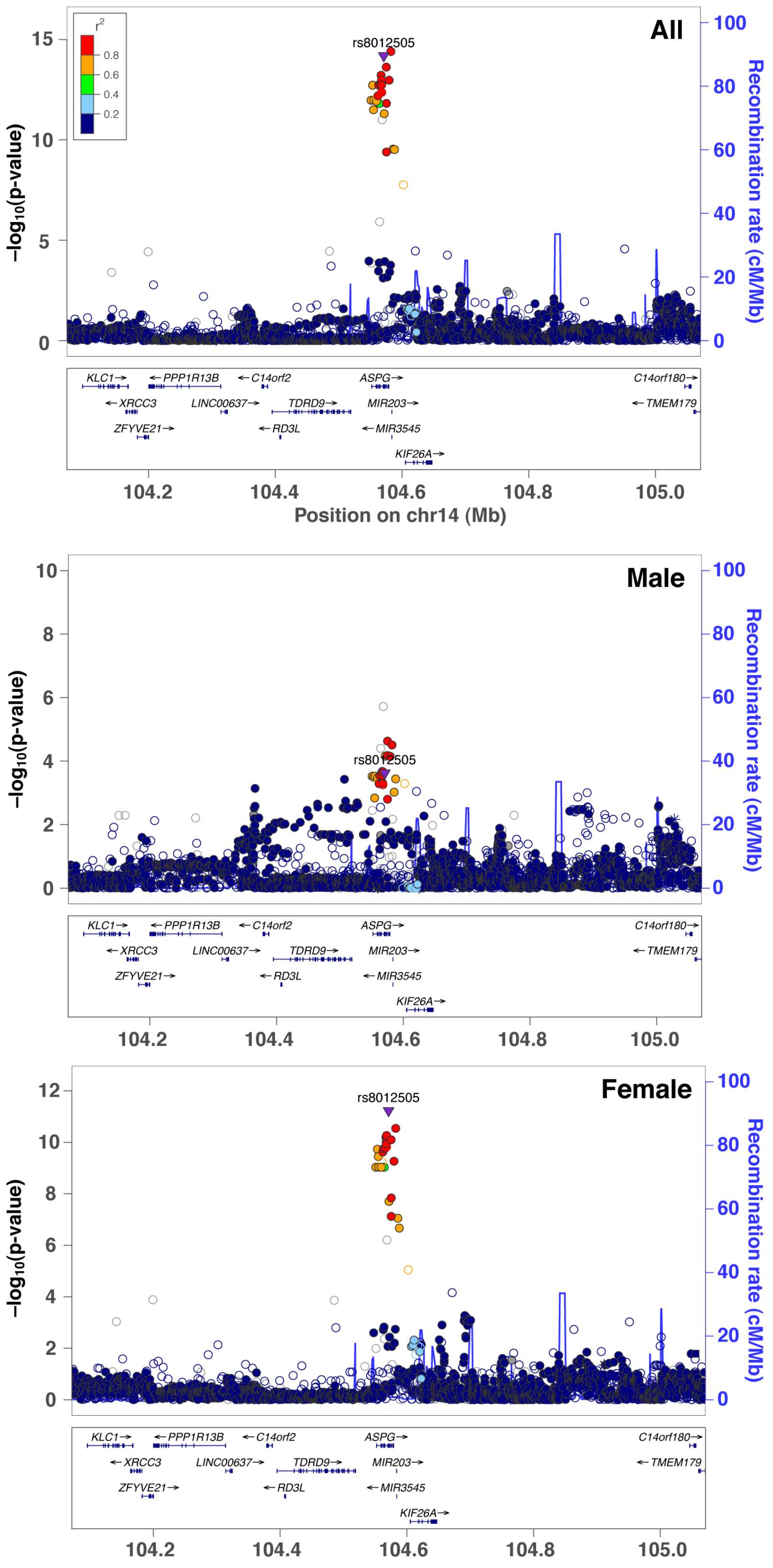

(b) Phenylalanine rs118092776

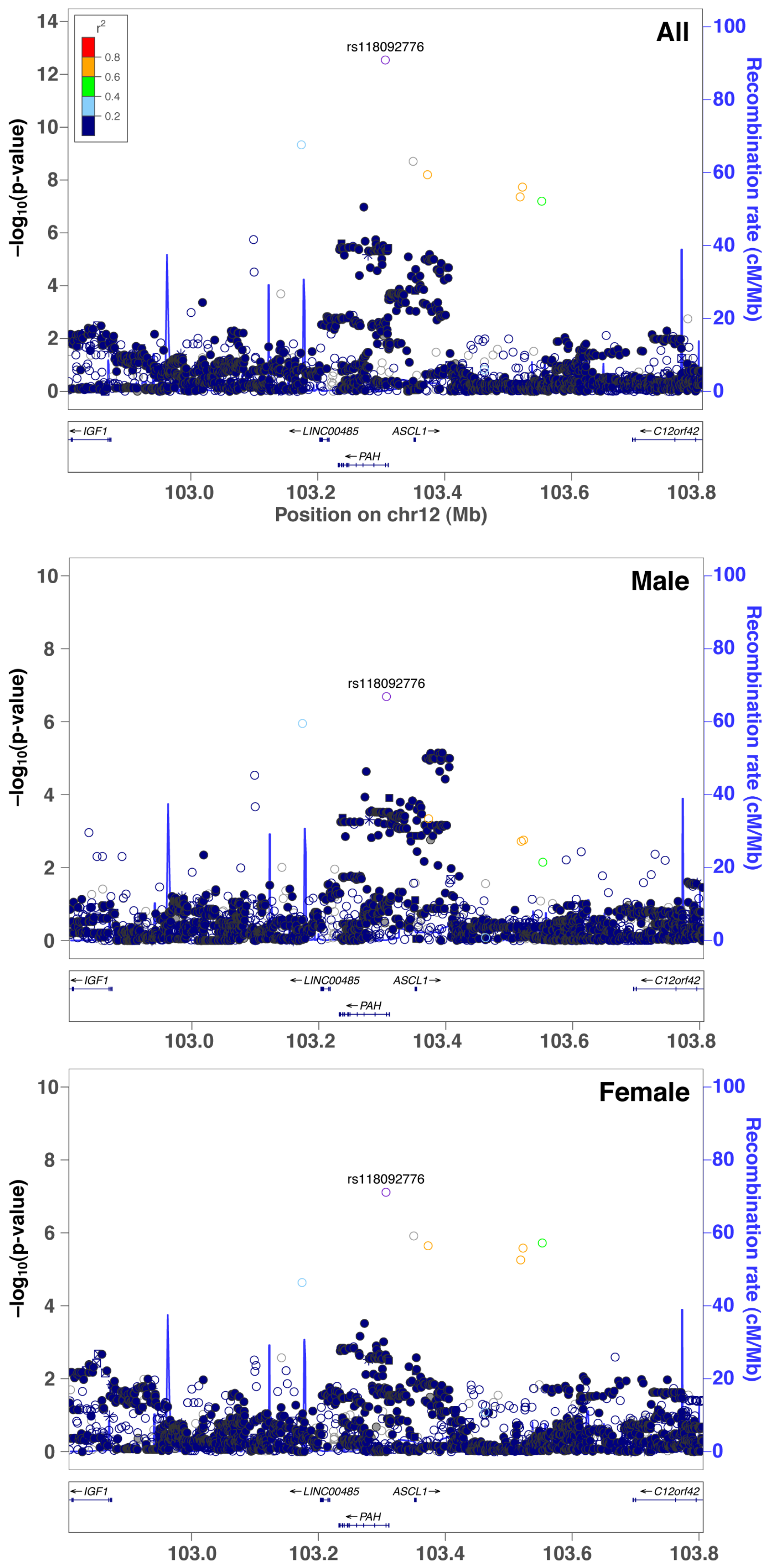

(c) Proline rs5747933

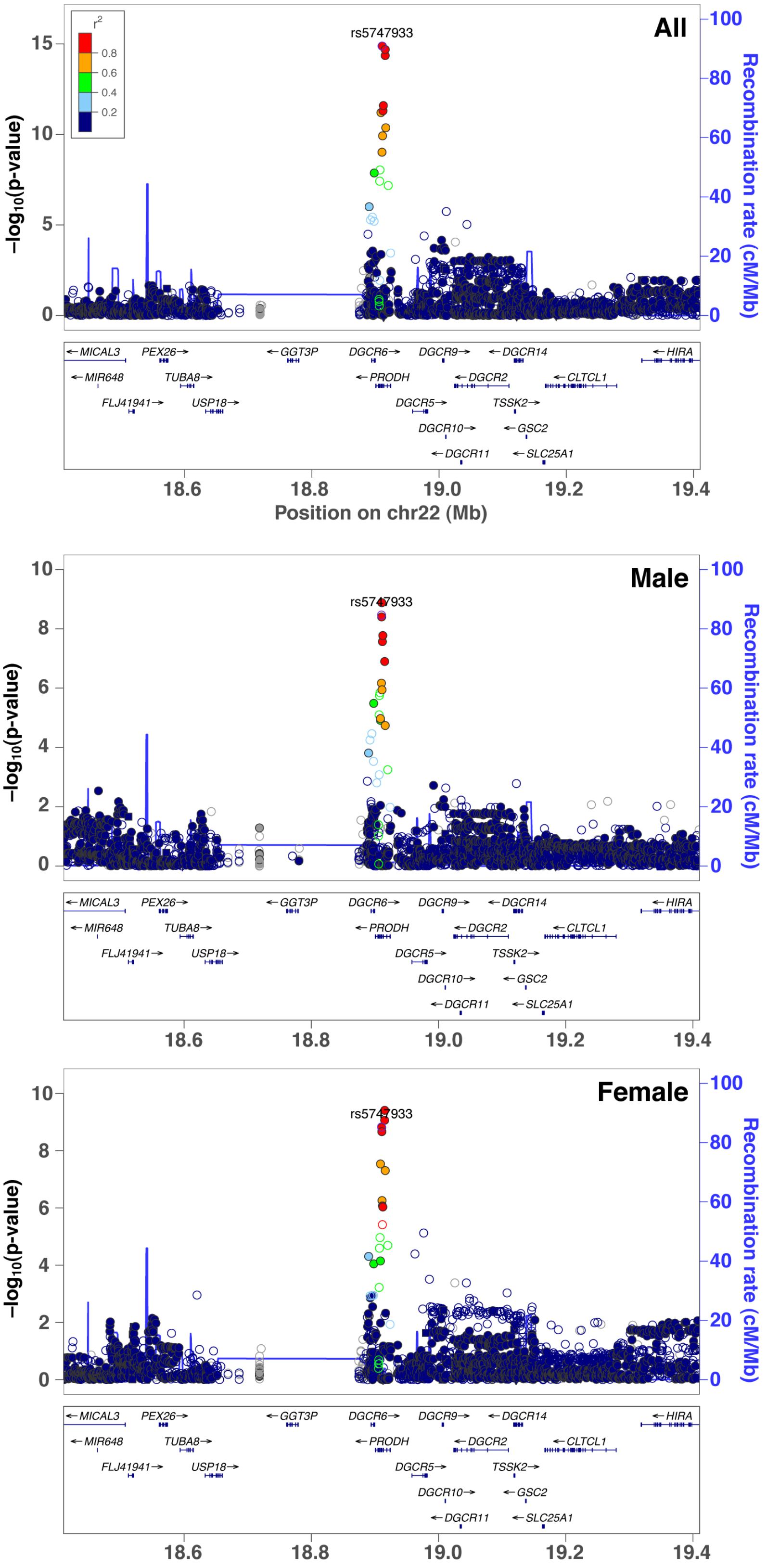

(d)

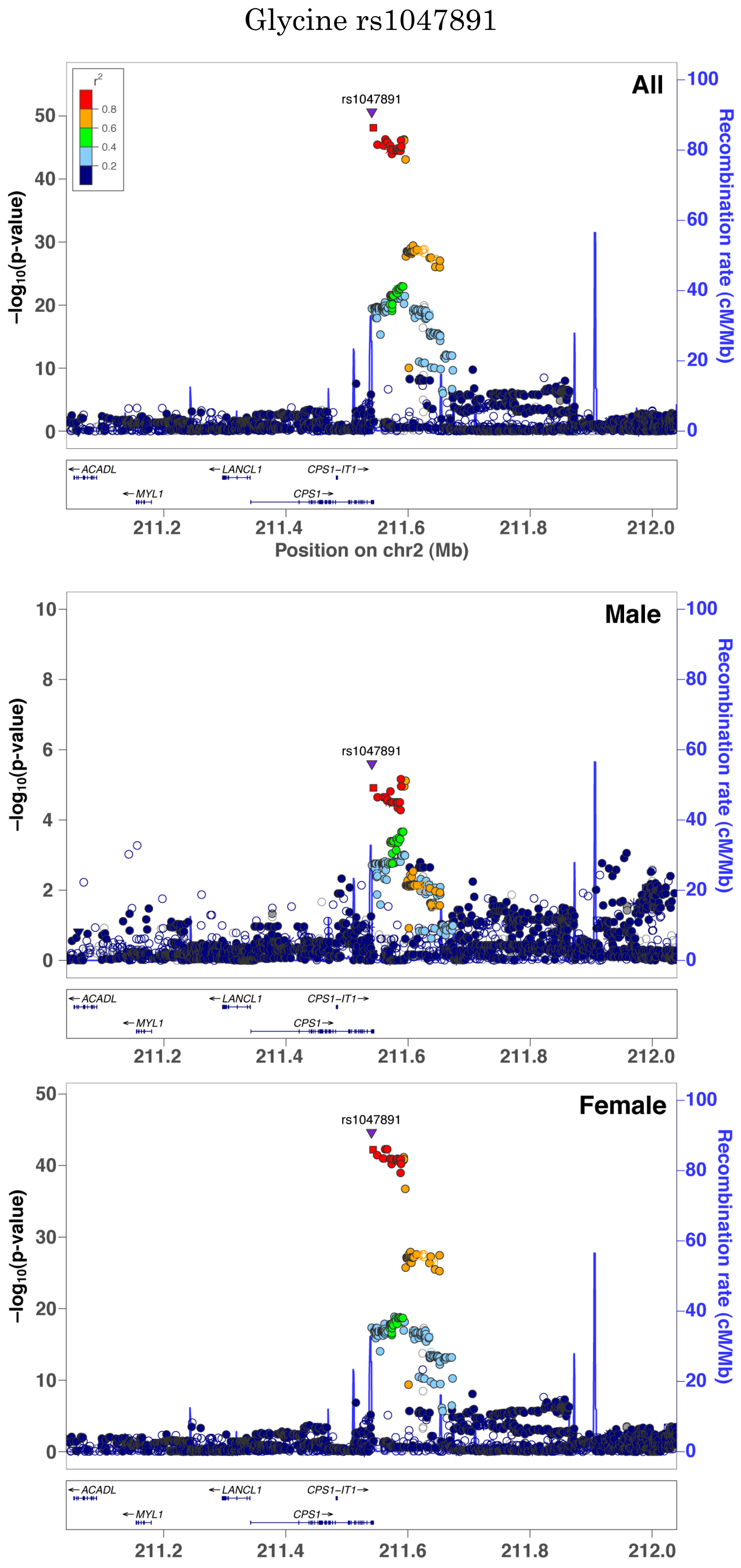
Glycine rs1047891
All
Male
Female
rs1047891
r2
0.8
0.6
0.4
0.2
−log10(p-value)
Recombination rate (cM/Mb)
ACADL
MYL1
LANCL1
CPS1−IT1
CPS1
211.2
211.4
211.6
211.8
212.0
Position on chr2 (Mb)

(e) Formate rs1801133

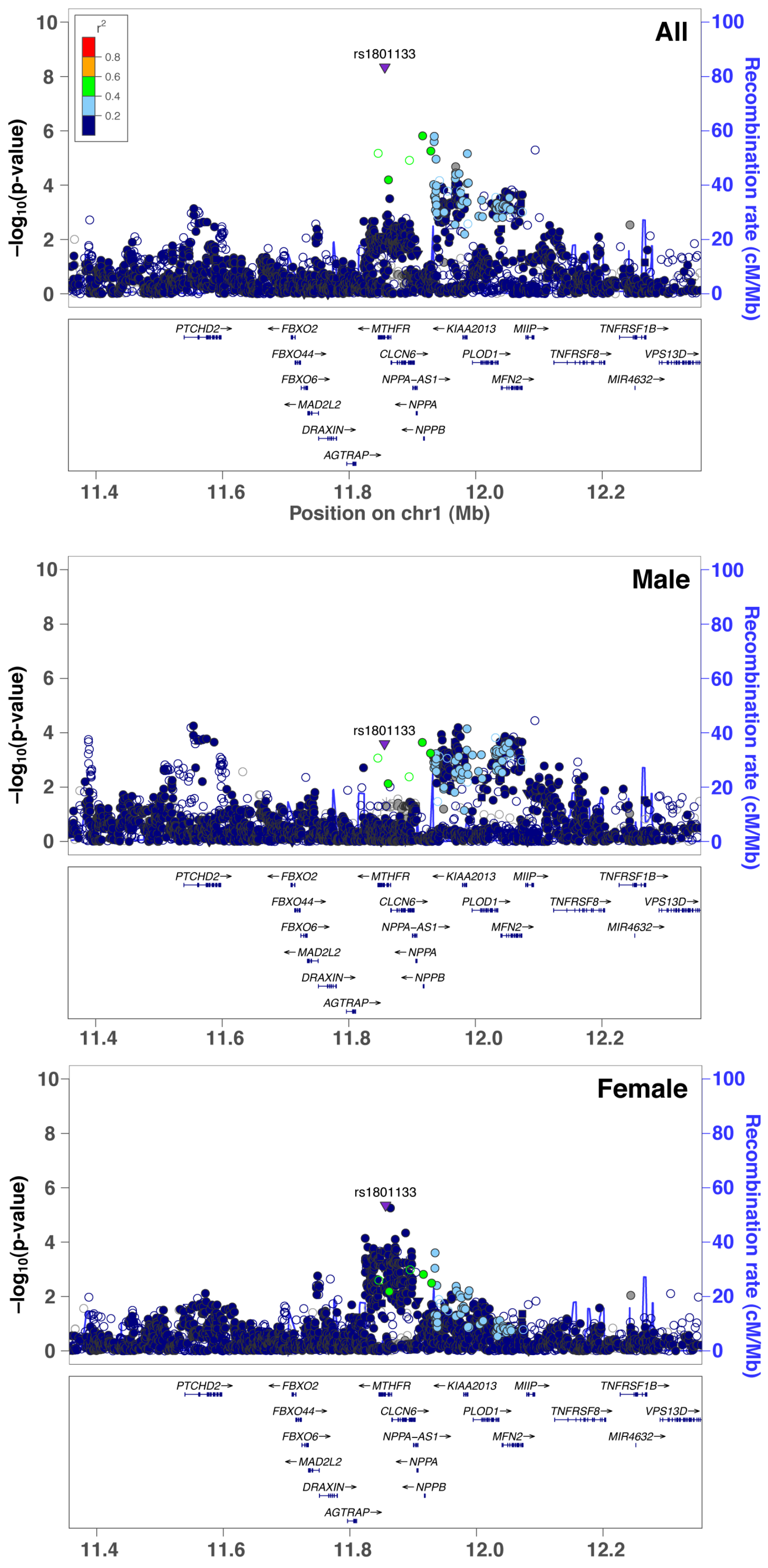

(f) Creatinine rs820336

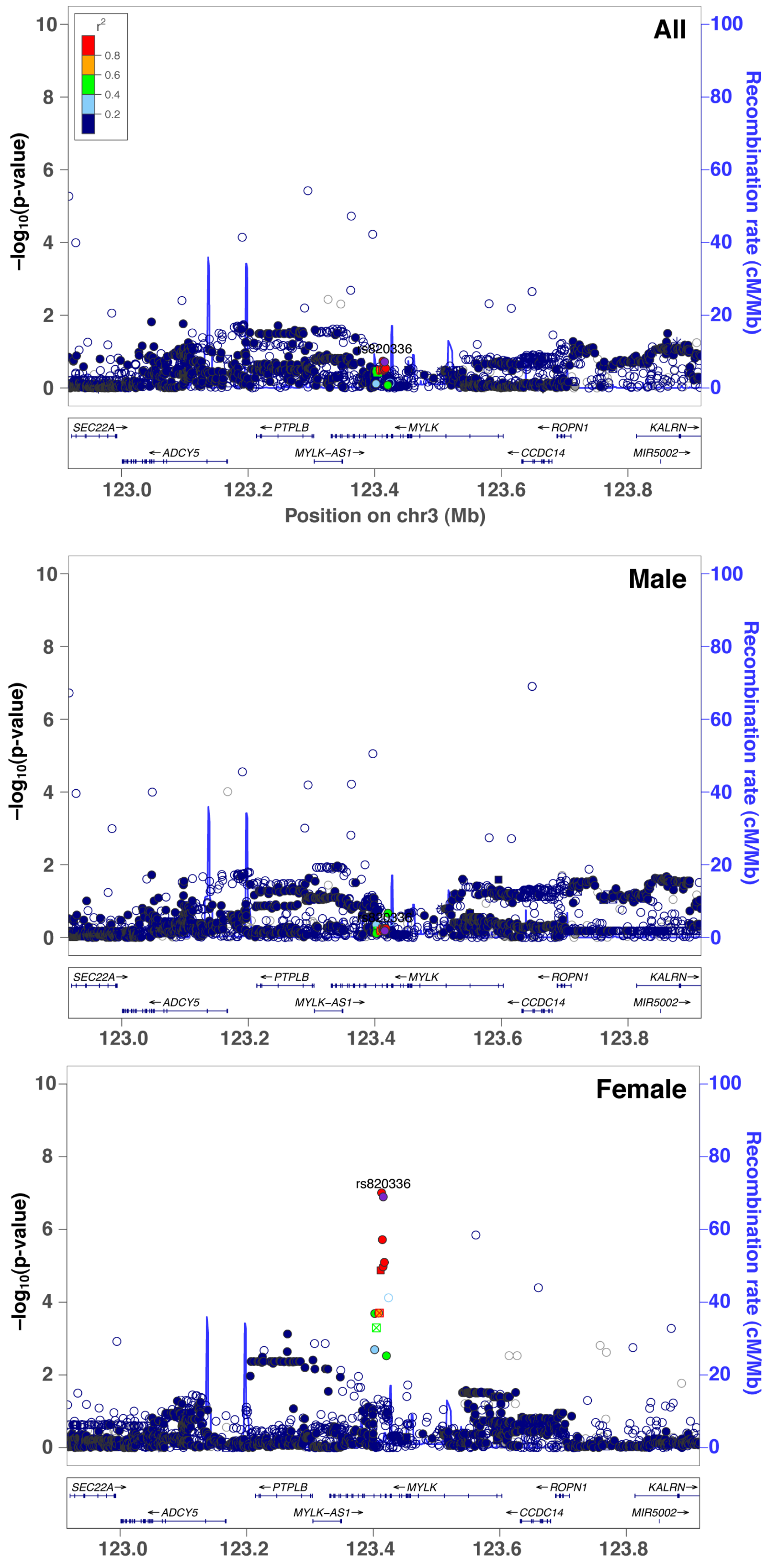

Supplementary Figure S1: Regional association plots for loci reported in this study. Statistical Significance of associated SNPs are plotted on the $-\log_{10}$(P-value) scale as a function of chromosomal position (NCBI 37). The identified causal SNP at each locus is shown in purple. Correlation of the causal SNP to other SNPs at each locus is shown on a scale from minimal (blue) to maximal (red). Estimate recombination rate are also shown.

a

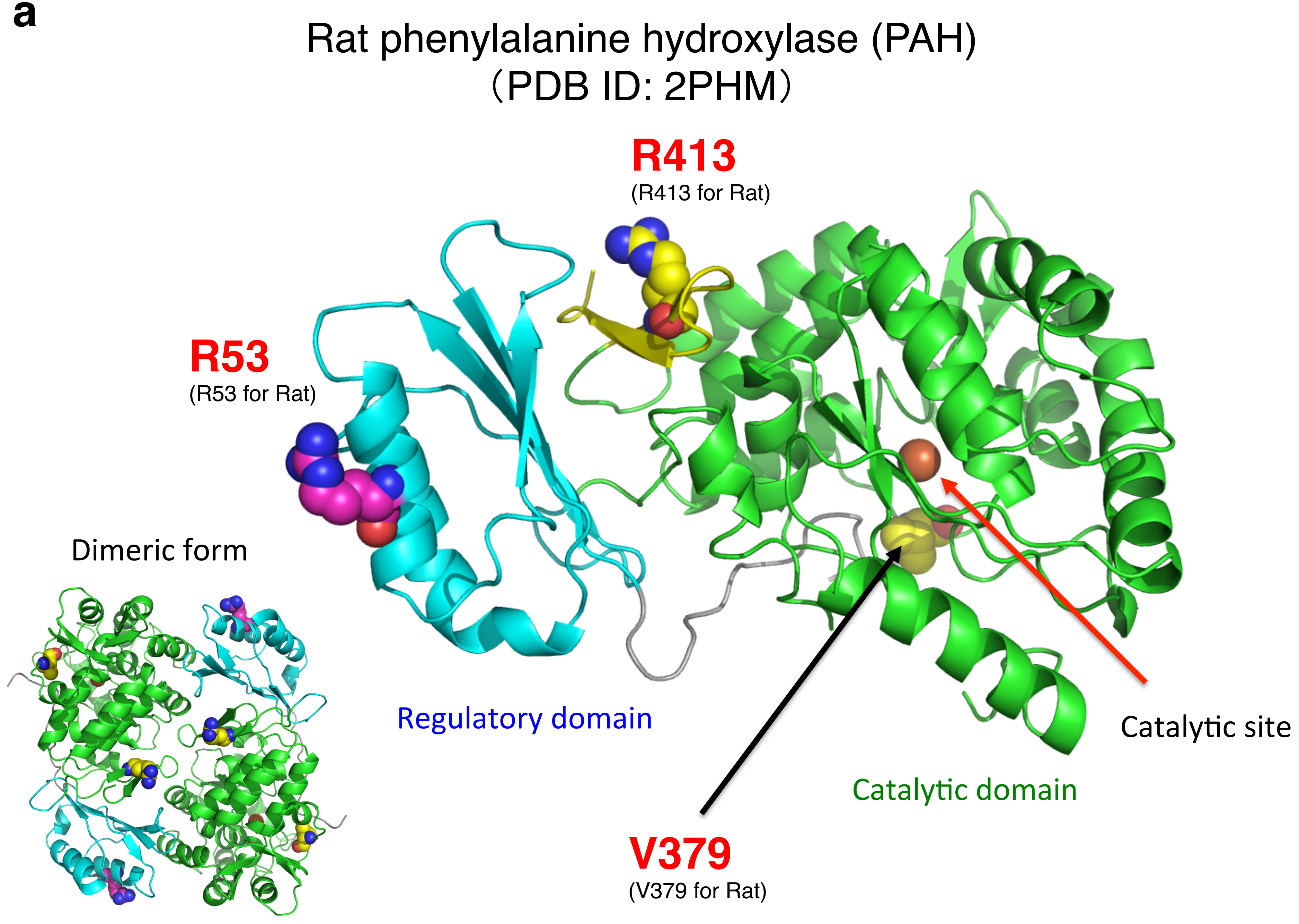
Rat phenylalanine hydroxylase (PAH)
(PDB ID: 2PHM)
R413
(R413 for Rat)
R53
(R53 for Rat)
Dimeric form
Regulatory domain
Catalytic site
Catalytic domain
V379
(V379 for Rat)

b

Human CPS1 C-terminal regulatory domain
（PDB ID:2YVQ）

E. Coli CPS (PDB ID: 1BXR)

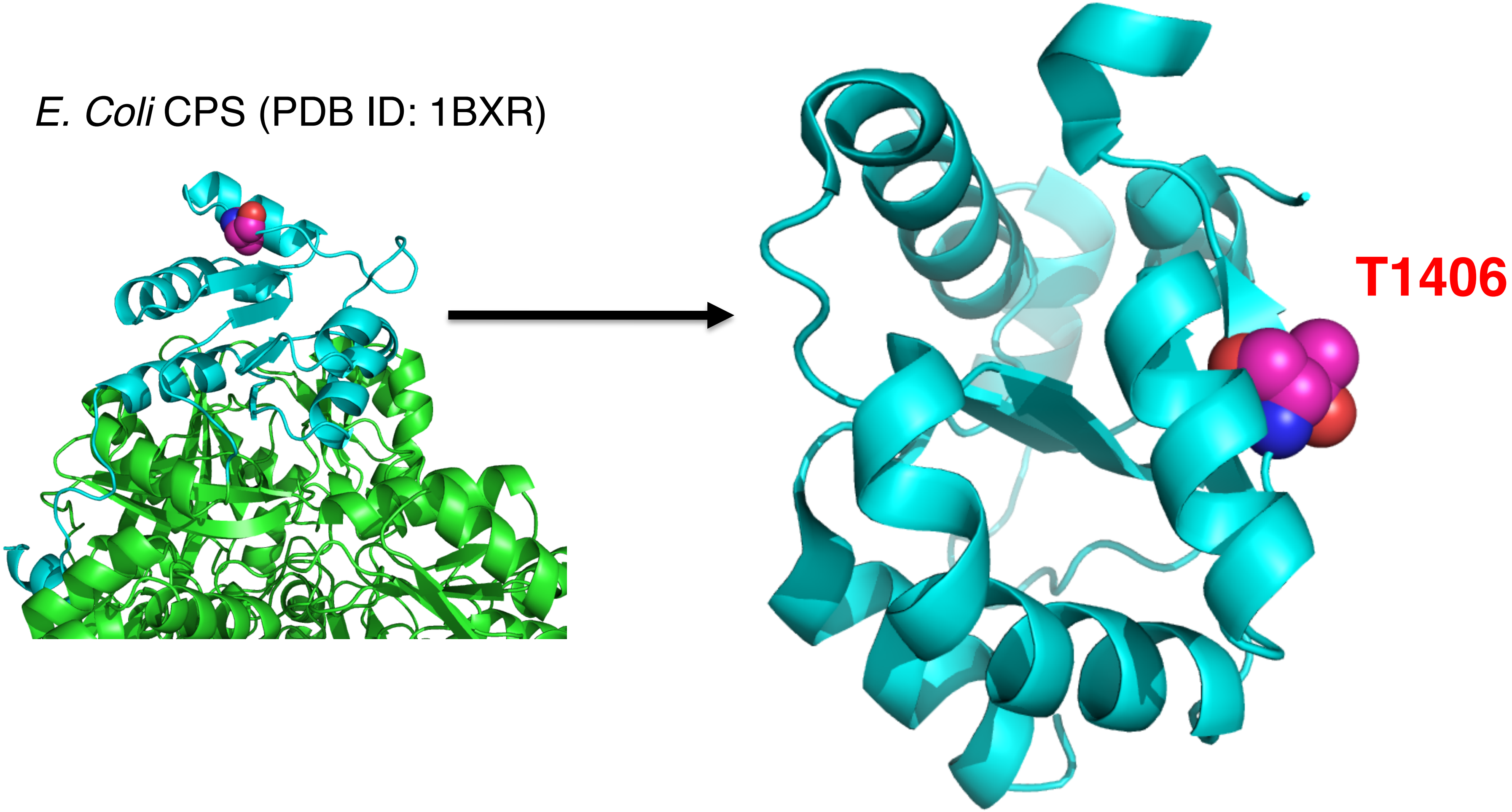

Supplementary Figure S2: (a) Structural characteristics of the non-synonymous variants in PAH. (b) Orientation of the regulatory domain relative to the catalytic domain in *E coli* CPS structure (PDB ID: 2BXR). All figures were made using the PyMOL Software Package (https://www.pymol.org/).

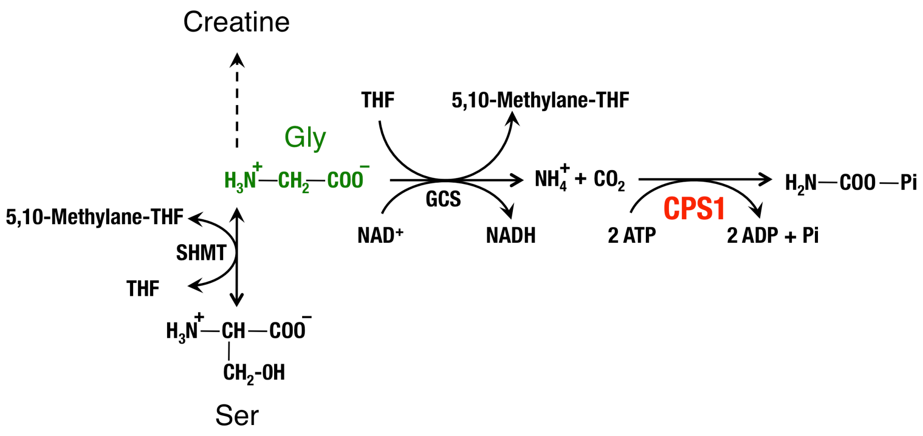

Supplementary Figure S3: Schematic representation of metabolite pathway involving glycine. CPS1 was shown in red. Glycine was shown in green.

rs820336 (MYLK)

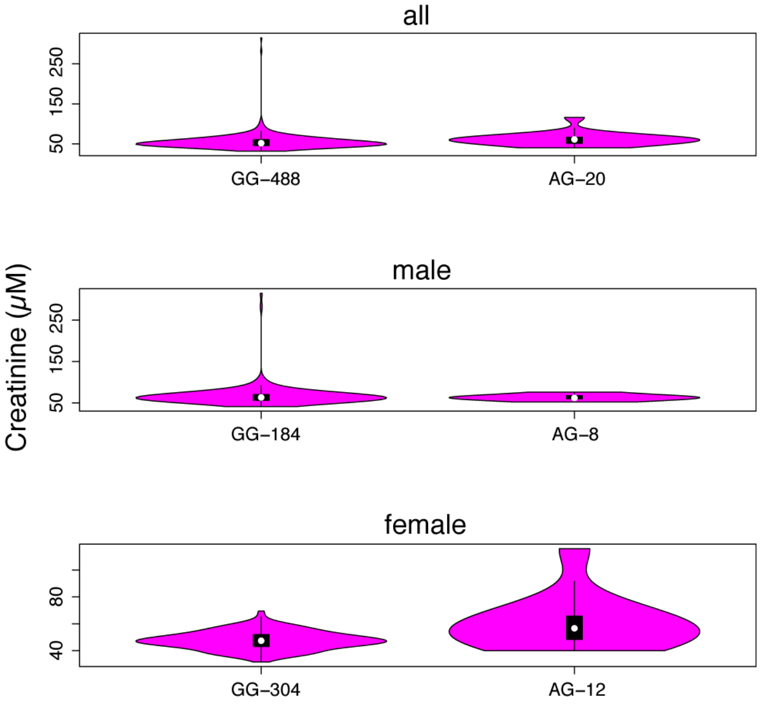

Supplementary Figure S4: Distribution of the plasma creatinine concentration across the genotype of a locus summarized in Supplementary Table S3b. The distribution was shown using a violin plot. This figure was made using R package.
